# Aldehyde Dehydrogenase Genes as Prospective Actionable Targets in Acute Myeloid Leukemia

**DOI:** 10.3390/genes14091807

**Published:** 2023-09-16

**Authors:** Garrett M. Dancik, Lokman Varisli, Veysel Tolan, Spiros Vlahopoulos

**Affiliations:** 1 Department of Computer Science, Eastern Connecticut State University, Willimantic, CT 06226, USA; 2 Department of Molecular Biology and Genetics, Science Faculty, Dicle University, Diyarbakir 21280, Turkey; lokman.varisli@dicle.edu.tr (L.V.); vtolan@dicle.edu.tr (V.T.); 3 First Department of Pediatrics, National and Kapodistrian University of Athens, Thivon & Levadeias 8, Goudi, 11527 Athens, Greece

**Keywords:** cancer bioinformatics, aldehyde dehydrogenase, biomarkers, gene expression, leukemia, myeloid, acute

## Abstract

It has been previously shown that the aldehyde dehydrogenase (*ALDH*) family member *ALDH1A1* has a significant association with acute myeloid leukemia (AML) patient risk group classification and that AML cells lacking *ALDH1A1* expression can be readily killed via chemotherapy. In the past, however, a redundancy between the activities of subgroup members of the ALDH family has hampered the search for conclusive evidence to address the role of specific *ALDH* genes. Here, we describe the bioinformatics evaluation of all nineteen member genes of the *ALDH* family as prospective actionable targets for the development of methods aimed to improve AML treatment. We implicate *ALDH1A1* in the development of recurrent AML, and we show that from the nineteen members of the *ALDH* family, *ALDH1A1* and *ALDH2* have the strongest association with AML patient risk group classification. Furthermore, we discover that the sum of the expression values for RNA from the genes, *ALDH1A1* and *ALDH2*, has a stronger association with AML patient risk group classification and survival than either one gene alone does. In conclusion, we identify *ALDH1A1* and *ALDH2* as prospective actionable targets for the treatment of AML in high-risk patients. Substances that inhibit both enzymatic activities constitute potentially effective pharmaceutics.

## 1. Introduction

The key aim in translational research for cancer treatment is to focus on the targeting mechanisms that allow malignant cells to resist cytotoxic chemotherapy. One general aspect of cancer cells’ resistance mechanisms to chemotherapy is the development of clones with an increased capacity to respond to cellular stress. Chemotherapy is administered to kill cancer cells, and it has been proven to be especially effective at killing cancer cells that operate error-prone systems of biomolecular synthesis and processing. However, the exposure of cancer cells to chemotherapy or any other cytotoxic conditions tends to select for clones that operate efficient cell stress adaptation mechanisms, which often act by accelerating the removal of mediators of cell death, or by preventing the accumulation of cytotoxic metabolites. The latter is particularly important for leukemia cells exposed to chemotherapy [1,2].

In cancer, in general, a disruption in tissue homeostasis represents a specific niche, perturbing the normal succession of inflammation–regeneration into a dysregulated process that enables the growth of malignant cell clones with a tumor-initiating potential [3,4]. This disruption is associated with changes in gene expression and the production of molecules that facilitate cancer progression, interfere with the function of the immune system [5], and deregulate the cellular response to oxidative stress [6]. Consequently, malignant cells increase the activity level of proteolytic systems such as the proteasome and the lysosome [7] that facilitate the recycling of biomolecules and activate antioxidant enzymatic systems for protection from chemotherapy. Thus, a cycle of gene regulation and metabolic adaptation occurs in cancer cells, enabling the survival of malignant clones that resist chemotherapy and may give rise to subclones with different biological properties.

Acute myeloid leukemia (AML) is no exception to this phenomenon: leukemia disrupts bone marrow function to enable the growth of malignant cell clones, which interfere with essential processes [8,9,10]. At the same time, the normal procedure of tissue recovery is disrupted, and cell phenotypes that are essential in re-establishing homeostasis, such as the anti-inflammatory macrophages, are enriched to support leukemia cells by suppressing the immune system and by assisting AML cells in meeting their metabolic demands, especially in respect to the mitochondria [11,12,13]. The disruption of normal function of the bone marrow niche is conducive to and ultimately exacerbates the aberrant production of cytokines that are essential in mediating the control of the inflammation–regeneration cascades; yet, in leukemia, they provide protection to malignant cells. Characteristically, AML stem cells (leukemia-initiating cells) show increased activity of the inflammatory transcription factor NFκB in contrast to that of normal hematopoietic stem cells [14,15,16].

Strong indications exist that an AML-negative disease course is associated with the deregulation of cellular responses to oxidative stress; the enzyme 8-oxoguanine DNA glycosylase1 (OGG1) mutant S326C that provides extended support to NFκB transcriptional activity was observed more frequently in patients who experienced AML relapse, and these patients exhibited a shorter relapse-free survival rate [17]. Under excessive oxidative stress, OGG1 turns from a DNA repair enzyme to a protein that augments proinflammatory NFκB transcriptional activity. S326C has a lower threshold of response to oxidative stress, and therefore, is expected to potentiate NFκB transcriptional activity for an extended time course. The condition of increased oxidative stress leads to the increased production of reactive aldehydes, which can be inactivated by enzymes such as aldehyde dehydrogenase ALDH1A1 [18]. In mitochondria, OGG1 Ser326Cys leads to increased reactive oxygen species [19]. Reactive oxygen species can lead to cell death via the generation of reactive aldehydes; the latter is inactivated by ALDH enzymes.

Even though normal stem cells may show the increased expression of molecules such as *ALDH1A1*, this expression is maintained via mechanisms that follow the homeostatic requirements of the host organism [20]. After exposure to chemotherapy, AML cells typically die, with the exception of those AML cells that inactivate chemical or biological mediators of cell death [21,22]. This death of AML cells, however, facilitates the growth of possibly slower cycling clones that possess more effective defense from mediators of cell death; in particular, surviving AML cell clones typically have increased protection from oxidant stress, such as intracellular regulators of thiol/disulfide levels, which are often augmented by the defective bone marrow microenvironment [23,24,25].

In this context, AML cell clones that survive chemotherapy can be expected to have, among other systems, an increased expression of enzymes that detoxify reactive aldehydes, which are molecules that cause cell death unless they are inactivated [26]. Therefore, the increased expression of *ALDH* enzymes could be expected to protect AML cells from chemotherapy and to enable those AML cell clones that overexpress a specific vital *ALDH* enzyme to survive chemotherapeutic treatment and to be able to grow and re-establish leukemia at a later time point. That time point of return of AML is expected to correspond to the relapse of the disease. The cell clones that reestablish AML and are resistant to the originally utilized drug treatment can be viewed as leukemia-initiating cells, or leukemia stem cells. In some settings of cancer development, during the initial stages of cancer progression, as soon as clinically detectable tumors have developed, cancer stem cells may be underrepresented due to the comparably faster growth of neoplastic cell clones that suppress quiescence and stem cell attributes. The clones that suppressed stem cell attributes may be readily killed using chemotherapy that is aimed at proliferating cells, or with proteostasis inhibitors that interfere with the capacity of cancer cells to remove massively accumulated misfolded proteins or cell death mediators [27,28]. The result is that the initially underrepresented clones of cancer cells that readily enter quiescence and express mediators of drug resistance survive after chemotherapy and can grow to give rise to disease recurrence. Such an event would be expected to trigger an AML relapse.

The clearest manifestation of cancer cell resistance mechanisms to chemotherapy can be found in patients who develop a recurrent neoplastic disease. An example of recurrent hematologic cancer is relapsed AML. For AML, it was previously hypothesized that ALDH enzymatic activity marks a positive outcome because a drop in activity was found in cancer patients when compared to that of healthy study volunteers; yet, soon after, in addition to identifying nonmalignant stem cells within some AML samples, high-level ALDH activity was also a marker of CD34^+^/CD38^−^ leukemic stem cells in some patients [29,30,31,32,33]. However, it was difficult to reach a definitive conclusion about the role of ALDH in leukemia development, as ALDH activity assays could not distinguish between different members of this protein family. However, patients with leukemia cells lacking *ALDH1A1* expression were later found to have a positive prognosis, and their leukemia cells could be killed using chemotherapy [34]. ALDH1A1 is a vital enzyme for AML cell detoxification from toxic aldehydes that arise after chemotherapy; although, it has a similar role in normal hematopoietic cells [35]. Additionally, we found previously that patients with a favorable prognosis generally expressed lower RNA levels from the gene that encodes ALDH1A1; furthermore, the high expression level of *ALDH1A1* RNA had a significant negative association with survival for AML patients [36].

Nevertheless, a redundancy between the activity of subgroup members of the ALDH family has hampered research efforts to find conclusive evidence for addressing the role of a specific *ALDH* gene in cancer. Herein, we analyzed publicly available data from leading AML studies with the objective of characterizing the relationship between the *ALDH* gene RNA expression with patients’ risk and survival in an effort to focus on therapeutically relevant findings, and specifically, on pharmaceutically actionable target genes.

## 2. Materials and Methods

We searched the literature for AML datasets containing RNA expression and risk or survival information. AML patient datasets were obtained from the Gene Expression Omnibus (GEO) [37], the Genomic Data Commons [38], and cBioPortal [39], and these were processed as described previously [36]. Briefly, RNA-seq data were processed using TMM (*edgeR* package) [40]; counts were converted to log counts per million (log CPM), and genes with <15 read counts across all samples were removed. Processed microarray datasets were downloaded from GEO. One dataset (BEAT AML) was retrieved using the *beatAML* package [41]. Beat AML samples were separated into patients with bone marrow aspirate (BMA) samples and patients with peripheral blood (PB) samples. Patients with both BMA and PB samples were excluded, and each sample type was analyzed separately. In the TARGET cohort, when comparing expression between primary and recurrent tumors, the patients with paired primary and recurrent tumor samples were analyzed separately from those with additional independent primary and recurrent tumor samples. Only primary tumors were included for all other TARGET analyses. For genes with multiple probes, the probe with the highest mean expression was used. Patients with risk assignments of ‘favorable’ or ‘low’ were considered ‘low risk’, while patients with ‘adverse’, ‘high’, or ‘poor’ risk levels were considered ‘high risk’. Samples labeled ‘intermediate’, ‘normal’, and ‘standard’ were excluded from the risk analysis. Association with risk was measured via the area under the receiver operating characteristics curve (AUC) using the ROCR package [42]. *p* values comparing groups were calculated using the two-sample *t*-test. For survival analysis, log rank *p* values were used to assess the statistical significance of survival curves. Confidence intervals for hazard ratios were calculated using the *confint* package in R, and the *forestplot* package was used to generate the forest plots. Expression data for LSC− and LSC+ samples [43], GEO Accession #GSE76008, were obtained using *shinyGEO* [44]. We calculated the LSC17 score using the same weights as described in [43].

## 3. Results

Our analysis is based on gene expression and clinical data from nine independent patient cohorts, with eight cohorts containing risk information (low- or high-risk; *N* = 860) and seven cohorts containing overall survival information (*N* = 1170). These datasets are summarized in Table 1. In these cohorts, age is positively associated with high risk in adult AML cohorts, while gender is not associated with risk (Table 2).

**Figure 1:**
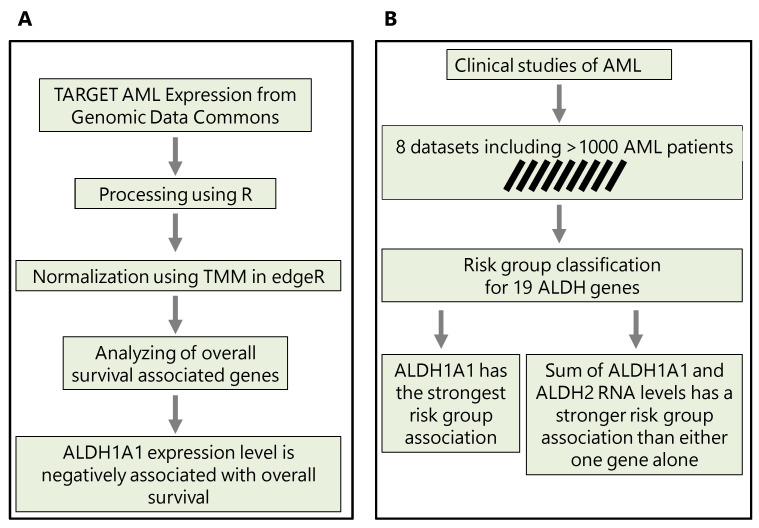
The methodological workflows conducted in this study. (**A**) Workflow to predict the implication of *ALDH1A1* expression in chemotherapy resistance. (**B**) Workflow to reveal the association of *ALDH1A1* expression level with risk groups and survival in AML patients.

In Section 3.1, we first compare the *ALDH1A1* expression between primary and recurrent tumors; in Section 3.2, we evaluate the association of all *ALDH1* genes with risk; in Section 3.3, we consider the top two genes and evaluate whether their combined expression is a better marker of risk and survival than either gene alone is. The workflows that we use and key findings are summarized in Figure 1.

### 3.1. Implication of RNA Expression from the ALDH1A1 Gene in AML Resistance to Chemotherapy

**Figure 2:**
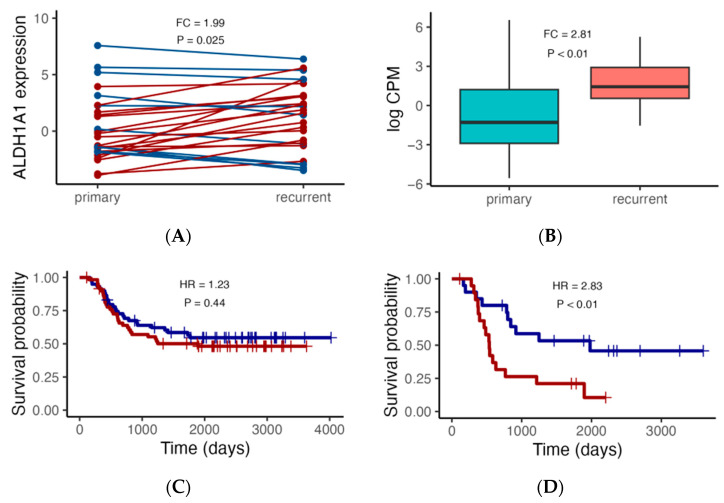
*ALDH1A1* RNA expression analysis in the TARGET cohort. (**A**) Comparison of *ALDH1A1* expression between paired primary and recurrent tumors (*N* = 27), with red lines indicating higher expression in recurrent tumors, and blue lines indicating lower expression in recurrent tumors. (**B**) Comparison of *ALDH1A1* expression in an additional set of independent primary (*N* = 92) and recurrent (*N* = 13) tumors. (**C**,**D**) Association of *ALDH1A1* RNA expression (blue: low; red: high) with patient overall survival. *p* values comparing primary and recurrent tumors were calculated using the paired and independent two-sample *t*-tests for (**A**) and (**B**), respectively; *p* values comparing survival curves for high and low expressors were calculated using the log rank test. HR: hazard ratio.

We first focused on *ALDH1A1* expression, since we previously demonstrated that, in pediatric AML, *ALDH1A1* gene RNA had a stronger association with risk group classification than the established biomarker *CALCRL* did [35]. We also focus initially on the TARGET cohort considering the 27 primary–recurrent paired samples and 105 additional independent patients with either primary (*N* = 92) or recurrent (*N* = 13) tumors. In the paired sample analysis, the expression level is higher in the recurrent AML samples (Figure 2A, FC = 1.99, *p* = 0.025). Similarly, when comparing *ALDH1A1* expression between additional primary and recurrent tumors, we also found that the *ALDH1A1* expression level is higher in recurrent tumors than it is in primary tumors (Figure 2B, FC = 2.81, *p* < 0.01). We then evaluated the association of *ALDH1A1* expression with overall survival and found that the hazard ratio is nearly twice as high in patients with recurrent tumors (HR = 2.38, *p* < 0.01) compared to that in patients with primary tumors (HR = 1.23, *p* = 0.44) (Figure 2C,D).

**Figure 3:**
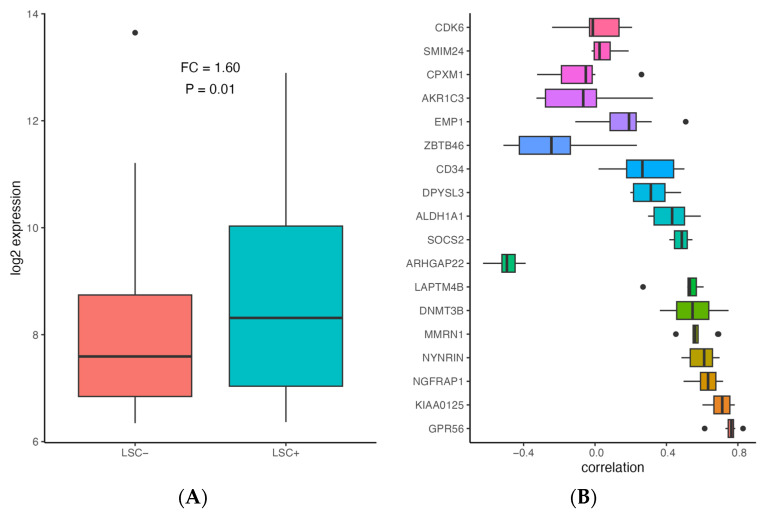
Association of *ALDH1A1* expression with stemness in AML. (**A**) Comparison of *ALDH1A1* expression between LSC− and LSC+ samples (*N* = 227) in GSE76008. (**B**) Correlation between gene expression and LSC17 score for *ALDH1A1* and LSC17 signature genes in 9 patient cohorts (described in Table 1). Genes are sorted based on median absolute correlation.

**Figure 4:**
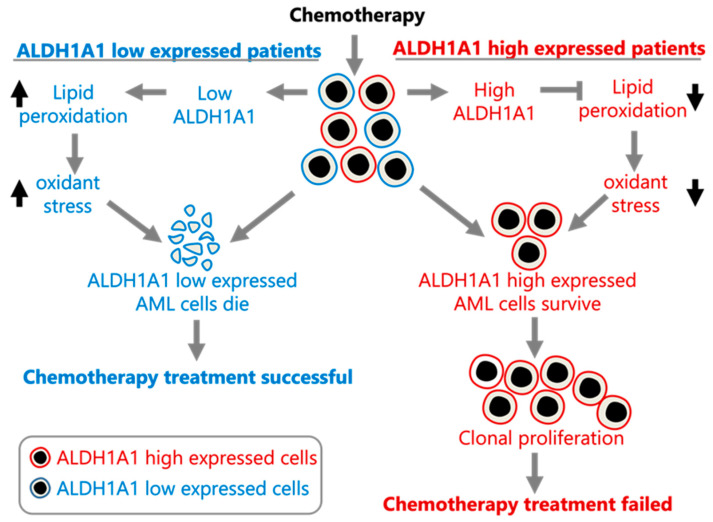
Proposed model of *ALDH1A1* contributing to chemoresistance in AML. The model was constructed using the data presented here and in [1].

In order to assess whether *ALDH1A1* is associated with leukemia stem cells (LSC), we compared *ALDH1A1* expression between 89 LSC− and 138 LSC+ cells using data from a previously published study that identified a 17-gene signature (LSC17) score associated with stemness and risk in AML [43]. *ALDH1A1* expression is up-regulated in LSC+ cells (FC = 1.6, *p* = 0.001) (Figure 3A), and it has a median correlation of 0.43 with the LSC17 score, which is comparable with the correlation of other signature genes in the patient cohorts we examined (Figure 3B). Our findings are consistent with *ALDH1A1* gene RNA expression involvement in the development of AML stem cell clones that are resistant to chemotherapy, which allows them to establish recurrent AML (Figure 4).

### 3.2. ALDH1A1 Is the ALDH Gene with the Strongest Risk Group Association

**Figure 5:**
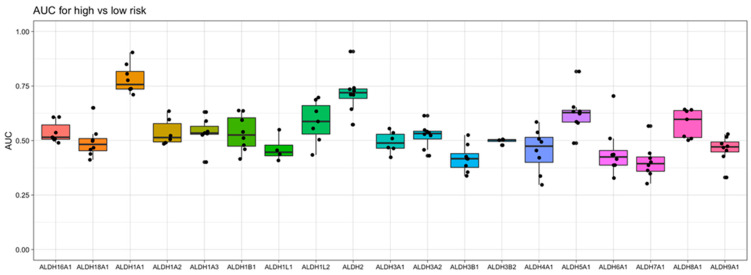
Evaluation of aldehyde dehydrogenase gene expression as a marker for risk in AML. The area under the “Receiver Operator Characteristic” curve (AUC) is used as a performance metric for how well gene expression separates low- and high- risk patients in 8 independent AML datasets. A value of 1 indicates perfect separation, while a value of 0.5 is the amount of separation expected by chance. Here, the *Y*-axis shows the values of AUC obtained with each gene, and the *X*-axis shows the *ALDH* genes examined.

To gain an understanding of the association of *ALDH1A1* gene expression with AML risk group classification and patient survival, we took into consideration nine independent datasets that were derived from six clinical studies of AML, which enrolled a total of over 1000 patients. When examining the relationship of *ALDH1A1* RNA expression level and risk group, we previously found that expression consistently differs across risk groups (*p* < 0.01) in the eight patient cohorts with risk information. In all cases, *ALDH1A1* gene expression level is the lowest in the “favorable” or “low” risk group, with an intermediate expression level in the intermediate risk group, and highest expression level in the high-risk group [36]. When we compared all nineteen *ALDH* genes for their association with risk group classification, we observed that *ALDH1A1* gives the strongest diagnostic separation between the patients with a favorable prognosis and the patients with an adverse prognosis (median AUC = 0.76). In fact, *ALDH1A1* had the seventh highest AUC value of all 20,330 genes profiled (in at least four datasets) in AML (Supporting Table S1). Interestingly, *ALDH2* gives the second strongest separation value (median AUC = 0.72) (Figure 5), though the *ALDH2* expression level was not higher in recurrent tumors (Supporting Figure S1) and is not associated with LSC cells (Supporting Figure S2), which contrasts *ALDH1A1* (see Section 3.1).

We also evaluated the association of *ALDH1A1* and *ALDH2* with the clinical parameters of age, gender, and FAB subtype. *ALDH1A1* is weakly positively correlated with age at diagnosis, with statistical significance (*p* < 0.05) in four patient cohorts, while *ALDH2* is statistically correlated (*p* < 0.05) in two patient cohorts. However, expression is not consistently associated with gender; the relationship between expression and gender has median AUC values of 0.53 for *ALDH1A1* and 0.45 for *ALDH2* (Supporting Table S2). Interestingly, *ALDH1A1* is consistently up-regulated in the M6 and M7 FAB subtypes, while no FAB subtype has a consistently high or low *ALDH2* expression level (Supporting Figures S3 and S4).

### 3.3. Combined RNA Expression Levels from the Genes ALDH1A1 and ALDH2 Have a Stronger Risk Group and Survival Association Than Either Gene Alone Does

**Figure 6:**
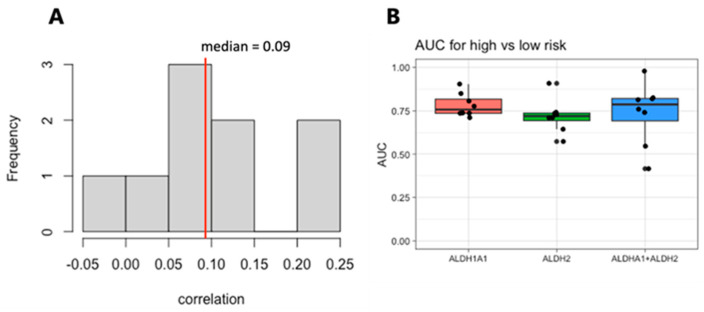
Correlation and analysis of combined *ALDH1A1* and *ALDH2* expression as a marker of risk in AML. (**A**). Histogram of the correlation between *ALDH1A1* and *ALDH2* expression in 9 independent patient cohorts. (**B**) AUC values for of *ALDH1A1*, *ALDH2*, and their combined expression for distinguishing patients with low- and high-risk tumors.

We next focused on the top two genes, *ALDH1A1* and *ALDH2*. The expression levels of these genes are weakly correlated within each dataset, with a median correlation of 0.09 (Figure 6A), suggesting that they may be independent markers of risk. We therefore evaluated the combined expressions of *ALDH1A1* and *ALDH2* for their association with risk and survival. The sum of the expression values for the RNA from the genes *ALDH1A1* and *ALDH2* shows a stronger risk group association based on the median than either one gene alone does (Figure 6B), but it also has a larger variance and is not stronger in all the cohorts.

**Figure 7:**
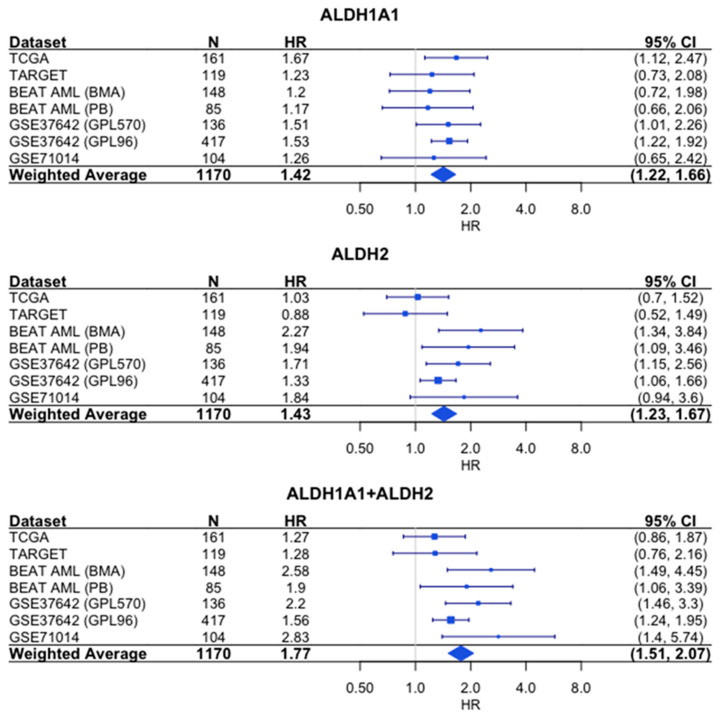
Association of RNA expression and survival for *ALDH1A1*, *ALDH2*, and their combined expression in AML. Each hazard ratio (HR) is calculated by comparing survival curves for patients with high expression level (≥median) to patients with low (<median) expression level. HR > 1 corresponds to patients with high expression level having a higher risk. For each individual cohort, the HR and 95% confidence interval (CI) are denoted by the blue rectangles and whiskers, respectively. The size of the blue rectangles is proportional to the precision of the HR estimate. For the weighted average, the diamond represents the 95% CI. *N* = number of patients.

Similarly, the sum of the expression values shows a stronger association with survival than either gene alone does based on the hazard ratios (HR = 1.77 for combined expression, which was compared to HR = 1.42 for *ALDH1A1* and HR = 1.43 for *ALDH2*) (Figure 7).

## 4. Discussion

Our analysis of the association of *ALDH1A1* RNA expression levels with recurrent AML leads to the suggestion that ALDH1A1 is involved in AML cell resistance to chemotherapy, potentially through the reduction of oxidative stress [18] and the survival of *ALDH1A1*-expressing leukemia stem cells. A model consistent with our findings is provided in Figure 4.

If this is true, then targeting ALDH1A1 in AML may sensitize tumor cells to chemotherapy. In fact, ALDH1A1 inhibition does sensitize colon, breast, pancreas, and ovarian cancer cells to chemotherapy [45,46,47,48], but further evidence is needed to conclusively determine whether this is also the case in AML. Additional evidence is also needed to confirm whether *ALDH1A1* expression drives tumor growth, and/or whether recurrent AML tumors have larger populations of cancer stem cells; though, we do see an association between *ALDH1A1* expression and stemness (Figure 3) based on a previously published AML stemness signature, where stemness was functionally defined [43]. While stemness can be defined functionally, stem cell associations are difficult to determine, since the only protein marker that can be consistently viewed as a leukemia stem cell marker is CD34 [49,50], and this marker also characterizes healthy hematopoietic stem cells. We also note that our analysis of recurrent AML is based on a single dataset with both primary and recurrent tumors, and it remains to be seen whether this result can be validated in additional prospective studies.

The association of *ALDH1A1* and *ALDH2* RNA expression levels with risk group classification and survival in patients with primary AML draws conclusions from a large sample size (>1000 patients) over nine independent cohorts (eight cohorts with risk information and eight cohorts with survival information), and therefore, they cannot be ignored. With up to 57% of AML patients having refractory AML or experienced a relapse or death within 12 months of diagnosis [51], what is important is the fact that both ALDH1A1 and ALDH2 enzymes can be targeted by molecules that inhibit both activities. Additionally, based on our survival and risk analyses, targeting these genes would likely provide the greatest benefit in high-risk, poor-outcome patients whose RNA expression of these genes is up-regulated. For targeting ALDH1A1 specifically, the greatest benefit would likely be in patients with M6 and M7 subtypes, which have the highest ALDH1A1 expression level (Supporting Figure S3) and the worst patient outcomes [52].

For AML class M6, a rare disease named acute erythroblastic leukemia that involves either undifferentiated or proerythroblast cells with no direct connection with ALDH exists in the literature; however, this connection is plausible because (a) during the course of erythroid differentiation, ALDH loses intensity, and (b) all reported erythroid cell lines that have been immortalized to date derive from the proerythroblast stage, with “evasion of oxidative stress-induced senescence” being the most represented pathway alteration [53]. In neoplasia, this type of pathway alteration is linked to the regulation of autophagy, mitochondria, oxidative stress, and NFκB [54].

Furthermore, oxidative stress resistance and NFκB-controlled gene expression characterize acute erythroblastic leukemia cell lines. In particular, NFκB prevents erythroid differentiation and also facilitates the expression of oncogene c-myc by preventing the execution of its apoptotic signals [4]. 4-Hydroxynonenal, a product of cellular lipid peroxidation, modulates c-myc and globin gene expression in K562 erythroleukemic cells [55]. As ALDH1A1 provides critical protection to cells from products of oxidative stress, and especially, 4-Hydroxynonenal, and is generally linked to stem and progenitor cells, it is highly likely to have a crucial role in the AML M6 disease course.

Although it is less consistent than *ALDH1A1*, *ALDH2* RNA expression is also associated with risk group classification, as shown in Figure 5. Although both enzymes oxidize aldehydes, ALDH1A1 is cytosolic-nuclear and accommodates larger molecules in the substrate binding site, and therefore, preferably oxidizes larger molecules; in contrast, ALDH2 is located in the mitochondria and has a small binding site, and therefore, metabolizes smaller aldehydes (acetaldehyde, formaldehyde, propionaldehyde, n-butyraldehyde, capronaldehyde, and heptaldehyde) [56]. Interestingly, *ALDH1A1* and *ALDH2* expression are weakly correlated (Figure 6A), and unlike *ALDH1A1*, *ALDH2* expression is not associated with recurrent tumors (Figure 1 vs. Supporting Figure S1), LSC+ cells (Figure 2 vs. Supporting Figure S2), or M6/M7 FAB subtypes (Supporting Figures S3 and S4), suggesting that *ALDH2* has a distinct relationship with risk in AML.

Future work should focus on whether targeting *ALDH* genes is an effective treatment strategy and whether *ALDH* expression drives patients’ outcomes. Indeed, our results do not show a causal relationship between *ALDH* expression and risk, but we do find a strong association, and this association is stronger than age in the cohorts we examined. We also note that while the sum of *ALDH1A1* and *ALDH2* expression shows a stronger relationship with risk, on average, than either gene alone does, the sum is not stronger in all the cohorts. Future work should, therefore, also compare treatment strategies that target either gene alone with treatment strategies that simultaneously target both genes.

Treating refractory and relapsed AML is a challenge, but efforts are underway to target genetic mutations such as FLT3-ITD and IDH1/IDH2 [57]. ALDH1A1 and ALDH2 can also be targeted. One molecule that targets ALDH1A1 is disulfiram, a substance approved for the maintenance of abstinence from alcohol. Disulfiram has been shown to target AML stem cells in cell lines and in primary AML samples [58], and the evaluation of disulfiram in combination with chemotherapy has shown promise in treating other cancers. In a phase II clinical trial for patients with metastatic non-small-cell lung cancer, patients receiving disulfiram in addition to cisplatin and vinorelbine chemotherapy had a modest improvement in terms of survival (10 months vs. 7.1 months) compared to that of patients receiving chemotherapy alone [59]. Furthermore, in a phase II clinical trial for patients with recurrent temozolomide (TMZ)-resistant glioblastoma, 14% of patients receiving disulfiram in addition to TMZ had a clinical benefit; though, the objective response rate was 0 [60]. Importantly, neither study selected patients on the basis of biomarkers that might predict disulfiram efficacy.

While disulfiram mainly inhibits ALDH1A1, in an organism, it is readily metabolized to substances that inhibit mainly ALDH2 [61]. Therefore, patients with high ALDH1A1 or ALDH2 activity levels would be expected to benefit from disulfiram treatment. The challenge would be to optimize the delivery of disulfiram to eradicate leukemia cell clones that escape the cytotoxic effects of chemotherapy.

Additionally, other molecules exist that target more than one member of the ALDH family. Some examples are diethylaminobenzaldehyde (DEAB), which can induce the expansion of normal human hematopoietic stem cells [62], and dimethyl ampalthiolester (DIMATE), which can eradicate leukemia stem cells, while sparing normal progenitors, both in vitro as well as in mouse xenografts of human AML cells [63]. Additional ALDH2 inhibitors include daidzin, an isoflavone found kudzu plant [64], and CVT-10216, which is derived from daidzin [65]. However, currently, only disulfiram is approved for clinical use, even though it needs to be repurposed for AML.

A possible undesirable side effect of disulfiram would be the induction of the expression of immunosuppressive molecules such as PD-L1, which could necessitate the additional use of this anti-PD1 treatment [66]. On the other hand, disulfiram when used either alone or combined with immunomodulating substances elicits the strong stimulation of components of the immune system [67] and has multiple intracellular targets that are able to elicit antitumor activity [68]. Nevertheless, it appears that ALDH1A1 is an important target of disulfiram, as the latter could inhibit breast tumor growth and tumorigenesis by purging ALDH+ cancer stem cells and activating T-cell immunity in xenografted mice, where it was shown that breast cancer cells expressing ALDH1A1 remodel myeloid-derived suppressor cells to enable cancer progression [69]. Thus, disulfiram has potent immunity-enhancing properties as well. The capacity of a drug to elicit multiple events in a cell is common and can be addressed via RNA profiling for the comprehensive evaluation of the complete picture. Nevertheless, certain facts in regard to the anti-neoplastic effects of anti-ALDH agents, and to disulfiram in particular, have already been established as it is reflected by the number of clinical trials that employ it [70].

These facts, taken together with our finding of the association of the high RNA expression levels of *ALDH1A1* and *ALDH2* in AML patients with a poor prognosis immediately suggest the development of a companion diagnostic that facilitates identifying the patients that are most likely to benefit from anti-ALDH and anti-PD1 treatments by measuring RNA expression. A guideline for companion diagnostics for oncology has been recently published by the US Food and Drug administration [71]. The development of a companion diagnostic for anti-ALDH treatment is expected to advance the use of ALDH inhibitors in precision oncology, and ultimately, integrate them into personalized AML treatment practices. The fact that both AML M6 and M7, which herein have the highest levels of *ALDH1A1* RNA expression level, have a very poor prognosis [72] necessitates follow-up research that increases the effectiveness of targeting AML cells.

## 5. Conclusions

Chemotherapy is one of the major weapons in the fight against cancer. However, in a significant proportion of patients, some sub-populations of cancer cells develop various molecular and/or cellular resistance mechanisms to these agents, and thereby, the treatment fails. Consequently, sensitive cells die depending on the chemotherapy treatment, whereas the resistant cell sub-populations survive and proliferate, and the disease recurs.

Our study suggests that the ALDH family member ALDH1A1 is one of the likely causes of recurrent AML, and also, that *ALDH1A1* is the *ALDH* gene with the highest association with patient risk group classification during primary AML. Drugs that inhibit both ALDH1A1 and ALDH2 enzymes are potential preclinical development candidates for AML, and our results suggest that the simultaneous targeting of both ALDH1A1 and ALDH2 will be more efficacious than targeting either enzyme alone.

## Figures and Tables

**Table 1:** Summary of patient cohorts.

Cohort	Description	*N* (Risk, Survival)	Data Availability	Reference (Pubmed ID)
TCGA	Adult patients with de novo AML	68, 161	Firehose	23634996
TARGET	Samples from pediatric patients from the NCI/COG TARGET-AML initiative, including 27 paired primary and recurrent tumors, and an additional 92 primary and 13 recurrent tumors	53, 119	GDC	26941285
BEAT AML (BMA)	Primary tumor samples from the BEAT AML program by bone marrow aspirate (BMA) or peripheral blood (PB)	113, 148	*beatAML* package	30333627
BEAT AML (PB)		63, 85		
GSE37642 (GPL570)	Independent patient cohorts from the German AMLCG 1999 trial	99, 136	GEO	23382473
GSE37642 (GPL96)		274, 417		
GSE6891 (Cohort #1)	De novo AML samples from patients under 60 years old	108, 0	GEO	18838472
GSE6891 (Cohort #2)		82, 0		
GSE71014	De novo AML samples from patients with normal karyotypes	0, 104	GEO	26517675

**Table 2:** Association of clinical parameters with risk. Association is quantified via AUC, which measures how well the parameter separates low- and high-risk patients. For age, AUC > 0.50 indicates a positive association with risk; for gender, AUC > 0.50 indicates that males are positively associated with high risk. * *p* < 0.05 by Wilcoxon Rank Sum test.

	Age		Gender	
Cohort	Mean (SD)	AUC	*N* (Female, Male)	AUC
TCGA	55.27 (16.14)	0.69 *	80, 93	0.60
TARGET	9.34 (6.09)	0.35	59, 60	0.66
BEAT AML (BMA)	55.47 (18.05)	0.61 *	74, 89	0.59*
BEAT AML (PB)	57.00 (18.95)	0.66 *	45, 50	0.42
GSE37642 (GPL570)	55.6 (14.62)	0.59	Not available	Not available
GSE37642 (GPL96)	54.72 (14.89)	0.65 *	Not available	Not available
GSE6891 (Cohort #1)	40.9 (12.2)	0.62 *	128, 119	0.51
GSE6891 (Cohort #2)	43.69 (12.11)	0.73 *	102, 112	0.48
GSE71014	Not available	Not available	Not available	Not available

## Data Availability

All data used in this study are available from the Genomic Data Commons (https://portal.gdc.cancer.gov/), Gene Expression Omnibus (https://www.ncbi.nlm.nih.gov/geo/), the Broad Institute Firehose (https://gdac.broadinstitute.org/), or the AMLBeatR package (https://github.com/radivot/AMLbeatR), as indicated in Table 1. All data was accessed on 30 March 2021.

## Supplementary Materials

The following supporting information can be downloaded at: https://www.mdpi.com/article/10.3390/genes14091807/s1. Table S1: All genes evaluated as markers of risk in AML; Table S2: Association of *ALDH1A1* and *ALDH2* expression with the covariates of age and gender; Figure S1: Comparison of *ALDH2* RNA expression between primary and recurrent tumors in the TARGET cohort; Figure S2: Comparison of *ALDH2* expression between LSC− and LSC+ samples (*N* = 227) in GSE76008; Figure S3: Association between *ALDH1A1* expression and FAB subtype; Figure S4: Association between *ALDH2* expression and FAB subtype.
