# Supplementary figures and images for: Aldehyde Dehydrogenase Genes as Prospective Actionable Targets in Acute Myeloid Leukemia
Source: Genes (Basel). 2023 Sep 16;14(9):1807. doi: 10.3390/genes14091807 (PMC10531322; doi:10.3390/genes14091807)

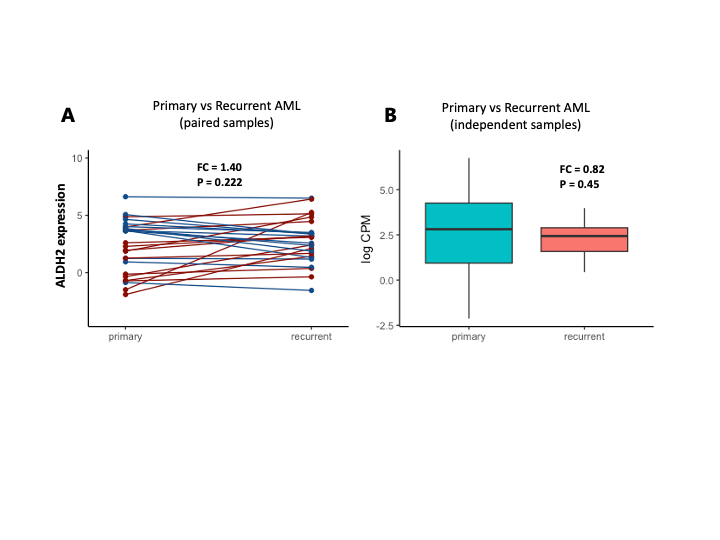

Supplement: Supplementary file 1 [file genes-14-01807-s001.zip › Figure S1.png]

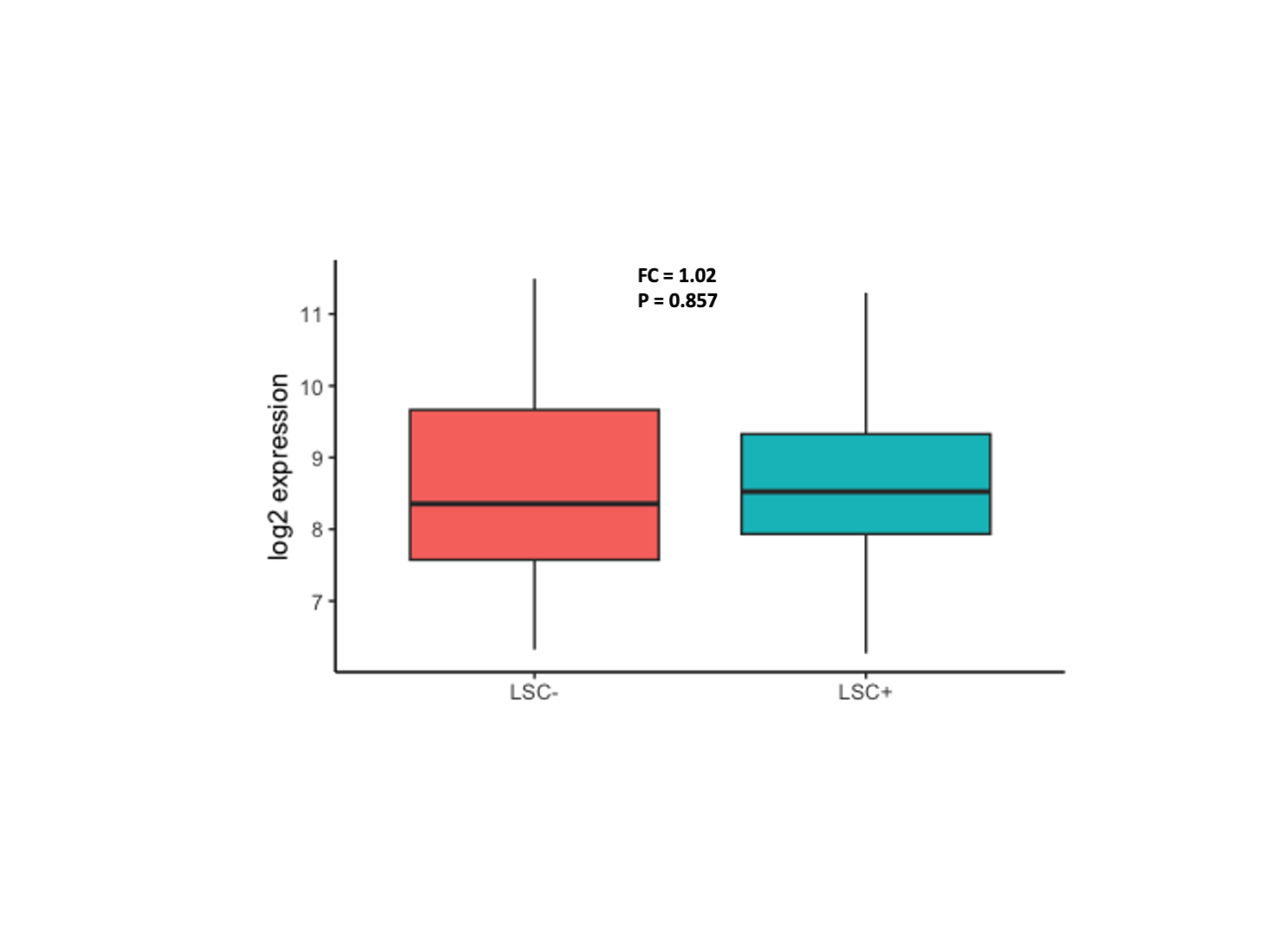

Supplement: Supplementary file 1 [file genes-14-01807-s001.zip › Figure S2.png]

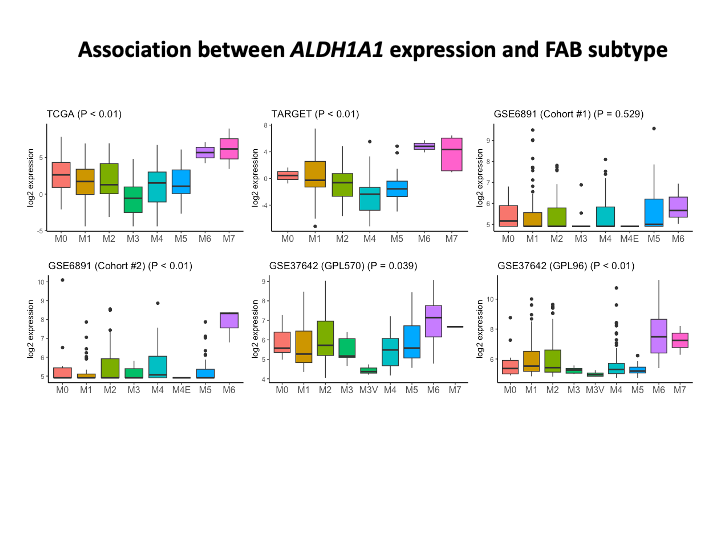

Supplement: Supplementary file 1 [file genes-14-01807-s001.zip › Figure S3.png]

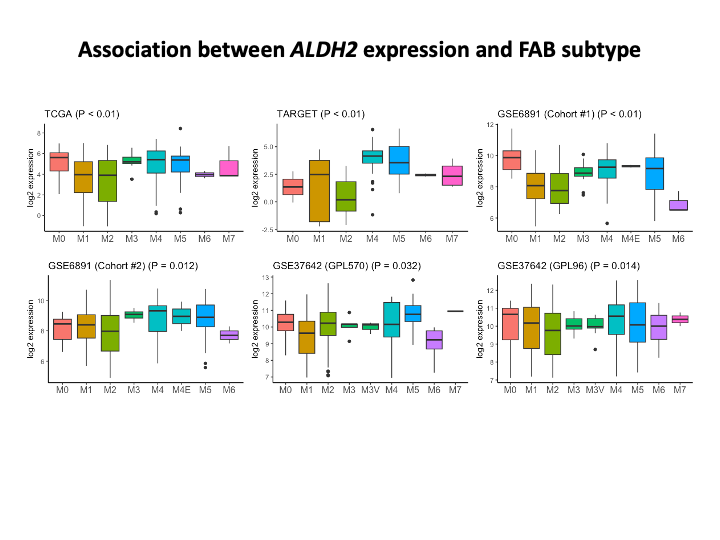

Supplement: Supplementary file 1 [file genes-14-01807-s001.zip › FigureS4.png]
